# Manganese superoxide dismutase mediates anoikis resistance and tumor metastasis in nasopharyngeal carcinoma

**DOI:** 10.18632/oncotarget.8717

**Published:** 2016-04-13

**Authors:** Shuai Li, Yuling Mao, Ti Zhou, Chuanghua Luo, Jinye Xie, Weiwei Qi, Zhonghan Yang, JianXing Ma, Guoquan Gao, Xia Yang

**Affiliations:** ^1^ Program of Molecular Medicine, Affiliated Guangzhou Women and Children's Hospital, Zhongshan School of Medicine, Sun Yat-Sen University, Guangzhou, China; ^2^ Department of Biochemistry, Guangzhou Medical University, Guangzhou, China; ^3^ Department of Physiology, University of Oklahoma, Health Sciences Center, Oklahoma City, USA; ^4^ China Key Laboratory of Tropical Disease Control (Sun Yat-Sen University), Ministry of Education, Guangzhou, China; ^5^ Key Laboratory of Functional Molecules from Marine Microorganisms (Sun Yat-Sen University), Department of Education of Guangdong Province, Guangzhou, China

**Keywords:** MnSOD, nasopharyngeal carcinoma, anoikis, reactive oxygen species, β-catenin

## Abstract

Metastatic cancer cells are able to survive the loss of attachment to the extracellular matrix (ECM) by developing resistance to anoikis, a specialized form of apoptosis. Here we investigated resistance to anoikis in nasopharyngeal carcinoma cells (NPC). When detached in culture, the highly metastatic S18 NPC cell line exhibited strong resistance to anoikis, as compared to the poorly metastatic S26 NPC cell line. With loss of attachment, S18 cells had lower levels of reactive oxygen species (ROS) and higher levels of manganese superoxide dismutase (MnSOD), an essential mitochondrial antioxidant enzyme. MnSOD knockdown increased the levels of ROS and diminished resistance to anoikis in S18 cells. Conversely, removal of reactive oxygen species (ROS) using NAC or overexpression of MnSOD in S26 cells induced resistance to anoikis. Blocking β-catenin through RNA interference down-regulated MnSOD expression and enhanced anoikis in S18 cells, while β-catenin overexpression enhanced MnSOD expression and suppressed anoikis in S26 cells. In addition, knockdown of MnSOD in S18 cells reduced colony formation *in vitro* and ameliorated lung metastasis *in vivo*. In patients with NPC, MnSOD expression was positively correlated with pathologic tumor stages and negatively correlated with overall survival. These results establish MnSOD as a key mediator of anoikis resistance and tumor metastasis and suggest that β-catenin/MnSOD could be a therapeutic target in NPC.

## INTRODUCTION

Nasopharyngeal carcinoma (NPC), derived from the epithelial lining of the nasopharrynx, is a commonly occurring cancer with the highest incidence of metastasis among head and neck cancers in southern China and Southeast Asia [1–4]. Because of its high radio-sensitivity, radiotherapy has been the main strategy for treatment of NPC. However, distant relapse remains the major cause of treatment failure in NPC [5], and the molecular mechanisms underlying NPC metastasis are poorly understood. In order to provide a basis for the development of novel therapeutics for NPC, it is crucial to obtain a better understanding of the molecular mechanisms used by cancer cells to facilitate their survival during metastasis.

While there are many abnormal features of metastatic cancer cells, resistance to anoikis is particularly interesting as it enables cell survival with loss of attachment to the extracellular matrix (ECM) [6, 7]. Metastatic cancer cells develop anoikis resistance by triggering several signaling pathways [6, 8, 9], including the production of reactive oxygen species (ROS) and activation of mitochondrial metabolism [10, 11]. Under physiological conditions, ROS are constantly generated as by-products of aerobic metabolism in the mitochondria [12, 13]. Consistent with the pro-anoikis abilities of ROS, treatment with antioxidants suppresses anoikis in breast cancer [10]. Moreover, energy deficiency (diminished ATP levels) caused by ECM detachment can reduce the viability of breast cancer cells [11].

ROS is a collective term for the chemical species that are formed as a result of incomplete reduction of oxygen. ROS include superoxide anion radical (O_2_^·−^), peroxyradical (ROO−), hydrogen peroxide (H_2_O_2_), singlet oxygen (^1^O_2_), perhydroxyl radical (HO_2_.), and the extremely reactive hydroxyl radical (.OH). The mitochondrial enzyme manganese superoxide dismutase (MnSOD) efficiently converts O_2_^·−^ to H_2_O_2_ and thereby critically changes mitochondrial destructive effects. Because superoxide arises primarily from the mitochondria, MnSOD plays a pivotal role in its detoxification [14, 15]. However, the role of MnSOD as a critical feature of highly metastatic NPC has not been elucidated. In this study, we assess the role of MnSOD in mediating the survival of ECM-detached NPC cells.

We provide evidence, both *in vitro* and *in vivo*, that up-regulation of MnSOD in a highly metastatic NPC cell line increases anoikis resistance after ECM detachment by decreasing mitochondrial O_2_^·−^ and accelerating hydrogen peroxide diminished. In addition, we show that the β-catenin pathway is robustly activated following a loss of ECM attachment. Activation of this pathway acts as a pro-survival signal, inducing a MnSOD-dependent cytoprotective response that is characterized by reduced mitochondrial O_2_^·−^ production and resistance to anoikis. Taken together, our findings suggest that β-catenin/MnSOD play central roles in mediating an antioxidant effect that enables cancer cells to survive and migrate to distant sites during metastasis.

## RESULTS

### Highly metastatic NPC cells have an advantage in anchorage-independent cell growth

**Figure 1:**
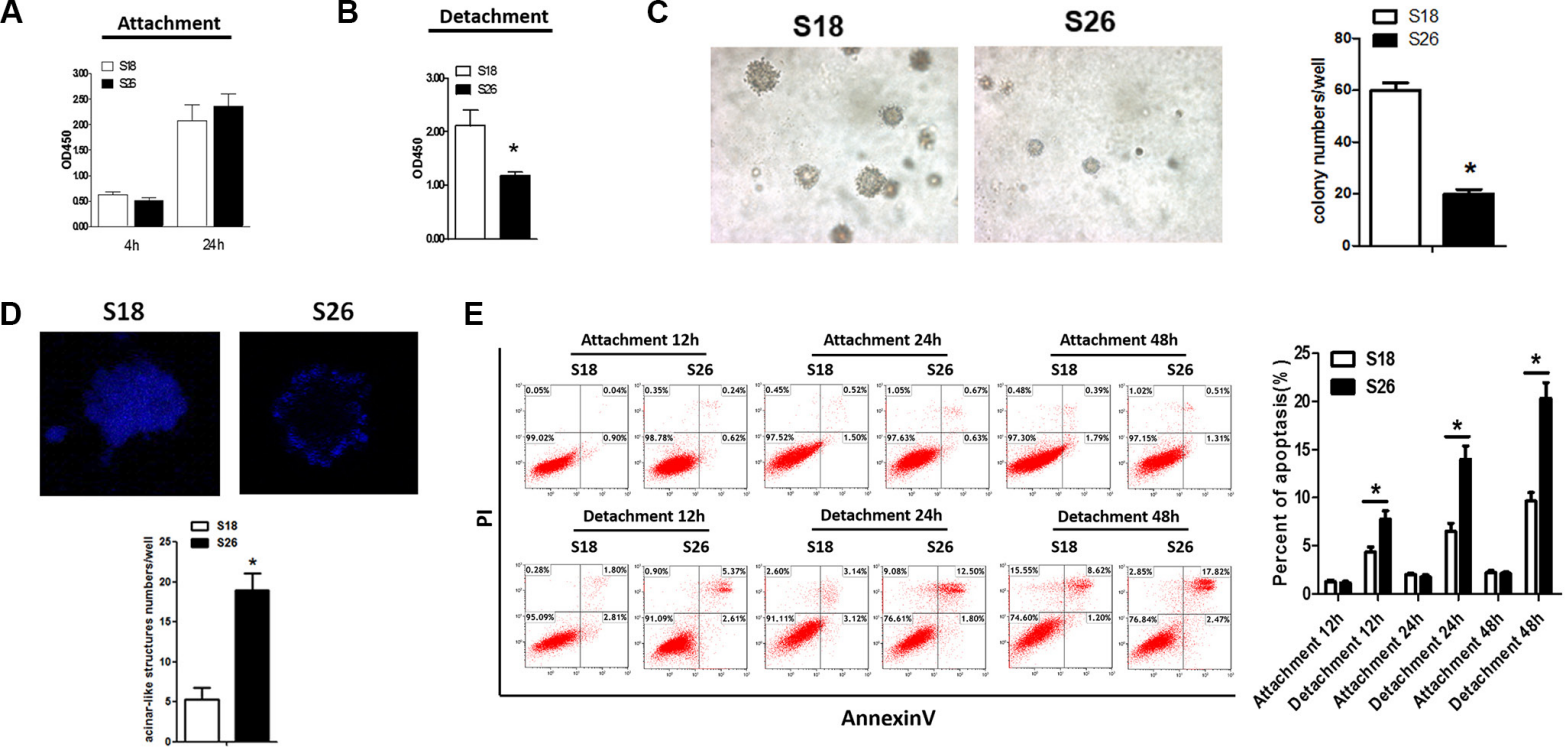
S18 cells exhibit enhanced anoikis resistance The viability of S18 and S26 cells under adherence (**A**) and suspended (**B**) conditions. (**C**) Colony numbers of S18 and S26 cells in soft agar. (**D**) Acini were generated using the indicated cell lines cultured in Matrigel for 12 days, and cells were stained with DAPI. Representative images are shown (× 200). *p < 0.05. (**E**) Flow cytometric analyses of S18 and S26 cell apoptosis under suspended conditions for 12 hours, 24 hours, and 48 hours. Values were plotted as mean ± S.D. *p < 0.05 compared with control group.

Malignant cells can often survive and grow without adhesion to the ECM making this growth a hallmark of the malignant phenotype [16, 17]. There is a well-established model to evaluate the relationship between metastatic potential and anoikis resistance [17, 18]. In this model, a highly metastatic cellular clone, S18, is compared with the poorly metastatic clone, S26. We tested clonogenesis of the S18 and S26 cells using a soft agar assay, and assessed cell viability and apoptosis of cells by CCK8 and AnnexinV/PI staining, respectively. Even though there was no obvious difference in cell viability between S18 and S26 cells under attached culture conditions (Figure 1A), S18 cells survived better in anchorage-independent cell growth conditions (Figure 1B–1C). When the NPC cells were cultured in a three-dimensional model to form clones in Matrigel, the clones generated from S18 cells formed more fully-filled structures when compared with S26 cells (Figure 1D). Moreover, anoikis was induced in S26 cells after 12, 24, and 48 hours of suspension conditions (Figure 1E). These observations demonstrate that cell viability is elevated with the aggressiveness of human NPC cells in response to matrix detachment.

### ROS induced by matrix detachment plays a critical role in NPC anoikis

**Figure 2:**
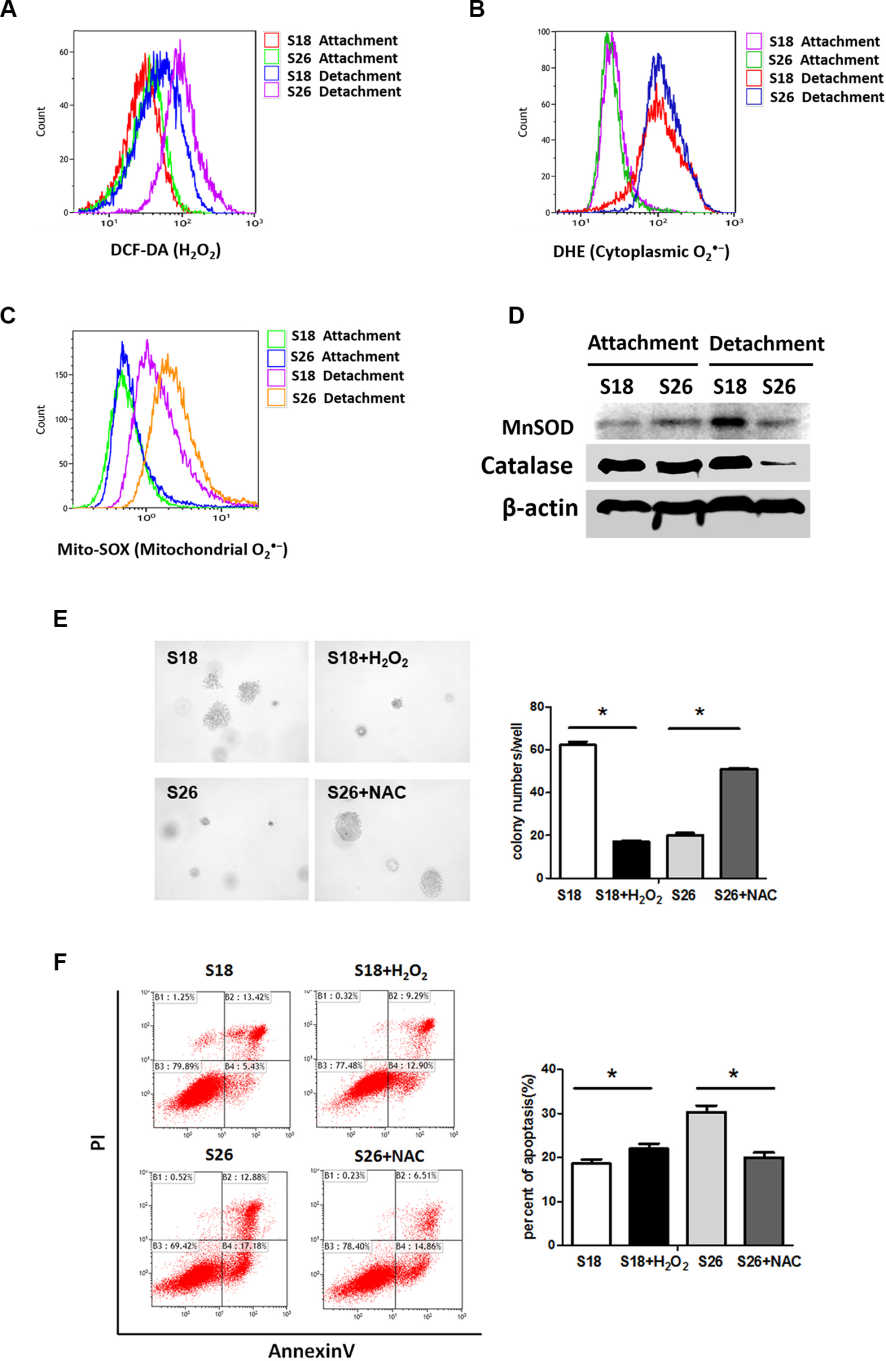
Enhanced ROS is critical for anoikis in NPC cells (**A**–**C**) S18 and S26 cells were incubated for 30 min in the presence of DCH-DA (10 μM), DHE (10 μM) or Mito-SOX (5 μM). Representative flow cytometry plots show the separate analysis of H_2_O_2_ (A), cytoplasmic O_2_^·−^ (B) and mitochondrial O_2_^·−^ (C) content in S18 and S26 cells under attachment and detachment cell growth condition after 24 hours. (**D**) The S18 and S26 cells were cultured in suspension conditions for 24 h, total cell lysates were subjected to western blot analyses performed to detect MnSOD and Catalase levels.(**E**) Quantification of soft agar colony numbers of S18 and S26 cells treated with H_2_O_2_ (30 μM) or NAC (1 mM). (**F**) Flow cytometric analyses of S18 cells and S26 cells by AnnexinV/PI under suspended conditions in the present of H_2_O_2_ (30 μM) or NAC (1 mM) as described. Values were plotted as mean ± S.D. *p < 0.05 compared with control cells.

Inadequate matrix attachment generates ROS and causes anoikis [10, 11]; therefore, we investigated the level of ROS in ECM-detached NPC cells. Since ROS are located in different subcellular compartments [19], we examined several different sub-cellular locations. There were no differences in DCF-DA fluorescence (for H_2_O_2_), DHE fluorescence (for Cytoplasmic O_2_^·−^) or Mito-SOX fluorescence (for mitochondrial O_2_^·−^) between S18 and S26 cells cultured on adherent plates. However, S18 cells had decreased H_2_O_2_ and mitochondrial O_2_^·−^ levels under detached conditions when compared with S26 cells (Figure 2A–2C). Since MnSOD and catalase are key antioxidant enzymes involved in scavenging ROS, we examined the protein levels of MnSOD and catalase in NPC cells grown in suspension. The MnSOD protein levels was up-regulated in S18 cells as compared to S26 cells. However, the protein levels of catalase was decreased in S26 cells when detached (Figure 2D). These results help understand why S18 cells have less O_2_^·−^ and H_2_O_2_. In addition, soft agar assays (Figure 2E) and Annexin/PI staining (Figure 2F) showed that treatment with 1 mM of an antioxidant compound N-acetyl-L-cysteine (NAC) stimulated the anchorage-independent growth in S26 cells, while treatment with 30 μM H_2_O_2_ decreased the anchorage-independent growth of S18 cells. These observations indicate that the level of ROS is inversely correlated with malign ant cell viability during the detachment process.

### MnSOD expression is elevated in detached highly metastatic NPC cells

**Figure 3:**
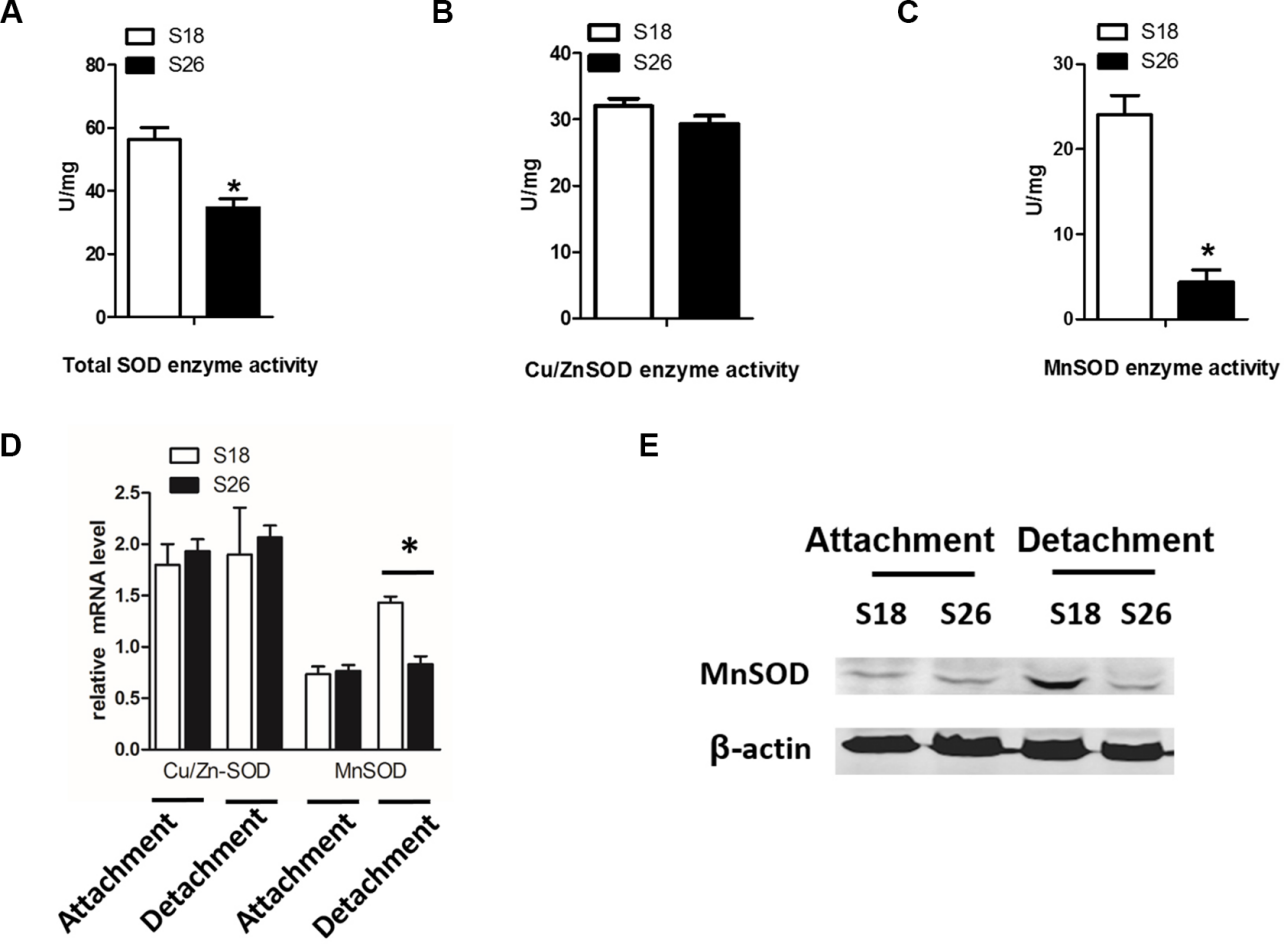
Matrix detachment induces MnSOD in high-metastasis S18 cells (**A**–**C**) S18 and S26 cells were cultured under detachment condition for 12 hours. Then, total SOD (A), Cu/Zn-SOD (B) and MnSOD (C) enzyme activity was measured using an SOD Assay Kit-WST. (**D**) Transcript levels of Cu/Zn-SOD and MnSOD in S18 and S26 cells under attachment and detachment. (**E**) MnSOD levels in S18 and S26 cells by western blot analyses. Values were plotted as mean ± S.D. *p < 0.05 compared with control.

We next investigated the molecular changes necessary for anoikis resistance and balancing the ROS concentrations. We examined Cu/ZnSOD activity as well as MnSOD activity in detached NPC cells. There was a difference in total SOD activity and MnSOD activity, but not Cu/ZnSOD activity, between S18 and S26 cells under detached conditions (Figure 3A–3C). When cells were cultured on adherent plates, there were no differences in Cu/Zn-SOD or MnSOD mRNA levels between S18 and S26 cells (Figure 3D). However, when cells were grown in suspension, both the mRNA and protein levels of MnSOD were upregulated in S18 cells as compared to S26 cells (Figure 3D–3E). These results suggest that MnSOD is necessary for the antioxidant defense in highly metastatic S18 cells and is critical for resistance to anoikis.

### MnSOD reduces ROS levels and anoikis-mediated cell death

**Figure 4:**
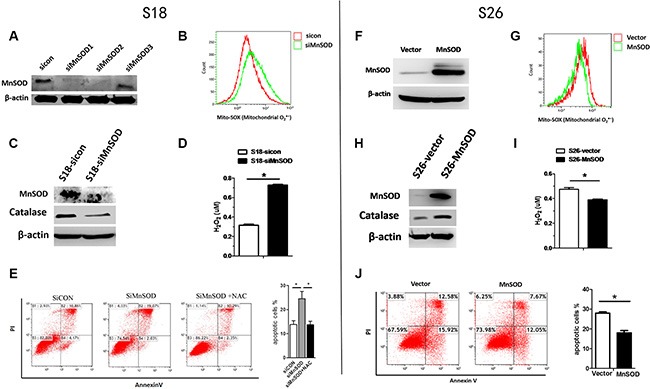
MnSOD confers resistance to anoikis S18 cells (**A**–**E**) were transfected with sicon or siMnSOD, S26 cells (**F**–**J**) were transfected with pcDNA3.1-vector or pcDNA3.1-MnSOD for 24 hours, after which they were cultured in poly-HEMA-coated plates under anchorage-independent conditions for 24 hours following which flow cytometry analysis for mitochondrial O_2_^·−^ (B, G), western blot analyses for catalase levels(C, H) were performed. H_2_O_2_ concentration (D, I) and apoptosis (E, J) were detected with Amplex^®^ Red Hydrogen Peroxide Kit and AnnexinV/PI, respectively. The data are presented as mean ± S.D. of triplicate determinations. For differences between two groups, the Students *t*-test was employed. *p < 0.05.

To further confirm the role of MnSOD in providing resistance to anoikis, we utilized MnSOD siRNA and a MnSOD overexpression plasmid. siRNA knockdown of MnSOD in S18 cells (Figure 4A) resulted in higher levels of Mito-SOX fluorescence (for mitochondria O_2_^·−^) (Figure 4B) and apoptosis (Figure 4E), corresponding to over 20% reduction of survival after 24 hours in suspended conditions and rendering these cells sensitive to NAC (Figure 4E). Therefore, MnSOD-mediated elimination of mitochondrial O_2_^·−^ in response to matrix detachment protects against anoikis. Conversely, overexpression of MnSOD in S26 cells (Figure 4F) reduced Mito-SOX fluorescence (Figure 4G) and decreased the percentage of apoptotic cells (Figure 4J), further supporting an essential role of MnSOD in anoikis resistance. However, SOD converts superoxide radical into hydrogen peroxide thus may establish an elevation of H_2_O_2._ Interestingly, subsequent analysis of the data demonstrated that MnSOD can promote protein expression level of catalase (Figure 4C, 4H) and H_2_O_2_ diminished (Figure 4D, 4I), which suggest that more MnSOD would be unlikely to yield more H_2_O_2_ in NPC cells.

### β-catenin signaling upregulated MnSOD and critical for anoikis resistance

**Figure 5:**
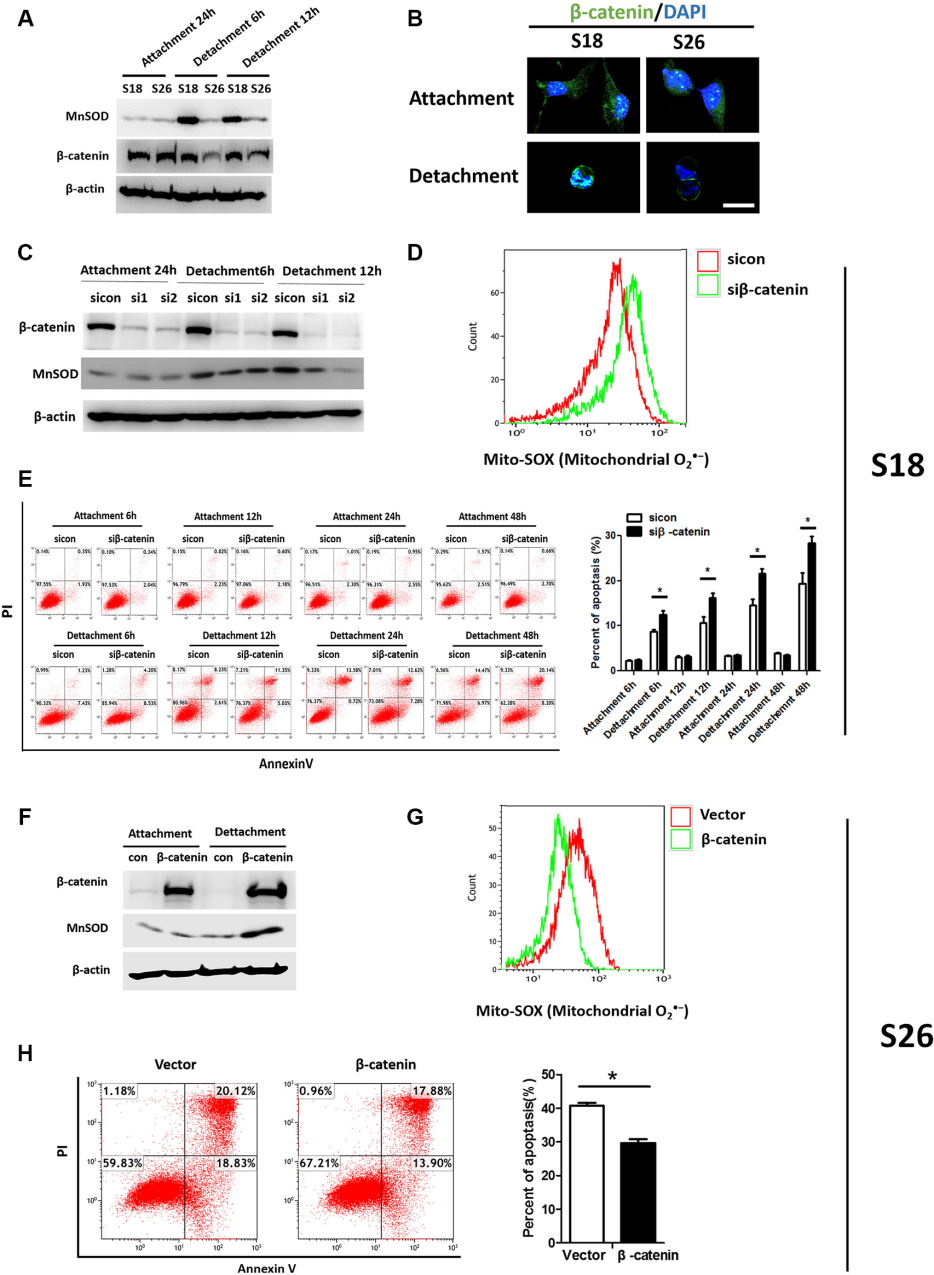
β-catenin signaling promotes anoikis resistance via MnSOD up-regulation (**A**) S18 cells were under adherent or suspended condition for 6 hours and 12 hours as indicated, total cell lysates, MnSOD, and β-catenin were subjected to western blotting. (**B**) Immunofluorescence staining of β-catenin. β-catenin in the cellular nucleus of S26 had a low intensity compared with that in the S18 after grown in suspension for 24 hours while there had no obvious difference of that when attachment cultured. (**C**) S18 cells were transfected with siRNA against β-catenin (siβ-catenin) or sicon. Immunoblots for indicated proteins were measured. (**D**) Flow cytometric analysis of mitochondrial O_2_^·−^ in S18 cells after cultured in suspension condition for 24 hours. (**E**) Flow cytometric analysis of cells by AnnexinV/PI under attachment or suspended conditions for 6 h,12 h, 24 h and 48 h as indicated. (**F**–**H**) S26 cells were transfected with empty vector or β-catenin for 24 hours and grown in attached or suspension condition for another 24 hours. Immunoblots of β-catenin (F), Flow cytometric analysis of mitochondrial O_2_^·−^ (G) and apoptosis rate (H) were shown. Values are plotted as mean ± S.D. *p < 0.05 compared with control.

Recently, several studies have suggested involvement of the Wnt/β-catenin pathway in ROS scavenging, and Wnt signaling is also known to function in tumor metastasis [20]. β-catenin transcriptionally regulates several antioxidant genes in response to oxidative stress [21]. Hence, we studied the putative connection between MnSOD and the canonical Wnt/β-catenin pathway. Interestingly, loss of β-catenin expression disrupted cell-cell adhesion in S26 cells and increased the nuclear accumulation of β-catenin in S18 cells after 6 hours of suspension (Figure 5A–5B). Knockdown of β-catenin in S18 cells reduced MnSOD protein levels in suspension (Figure 5C). Flow cytometric analysis confirmed that mitochondrial O_2_^·−^ levels were elevated in siβ-catenin cells compared with control cells following 12 hours of suspension (Figure 5D) and accompanied by increased anoikis (Figure 5E). Moreover, S26 cells overexpressing β-catenin had increased MnSOD protein levels (Figure 5F) and suppressed anoikis when in suspension (Figure 5H). On the contrary, Mito-SOX fluorescence intensity was reduced in S26 cells overexpressing β-catenin (Figure 5G). Together, these findings provide evidence for the involvement of β-catenin working upstream of MnSOD to promote anoikis resistance in metastatic NPC cells.

### Knockdown of MnSOD inhibits metastasis by promoting anoikis

**Figure 6:**
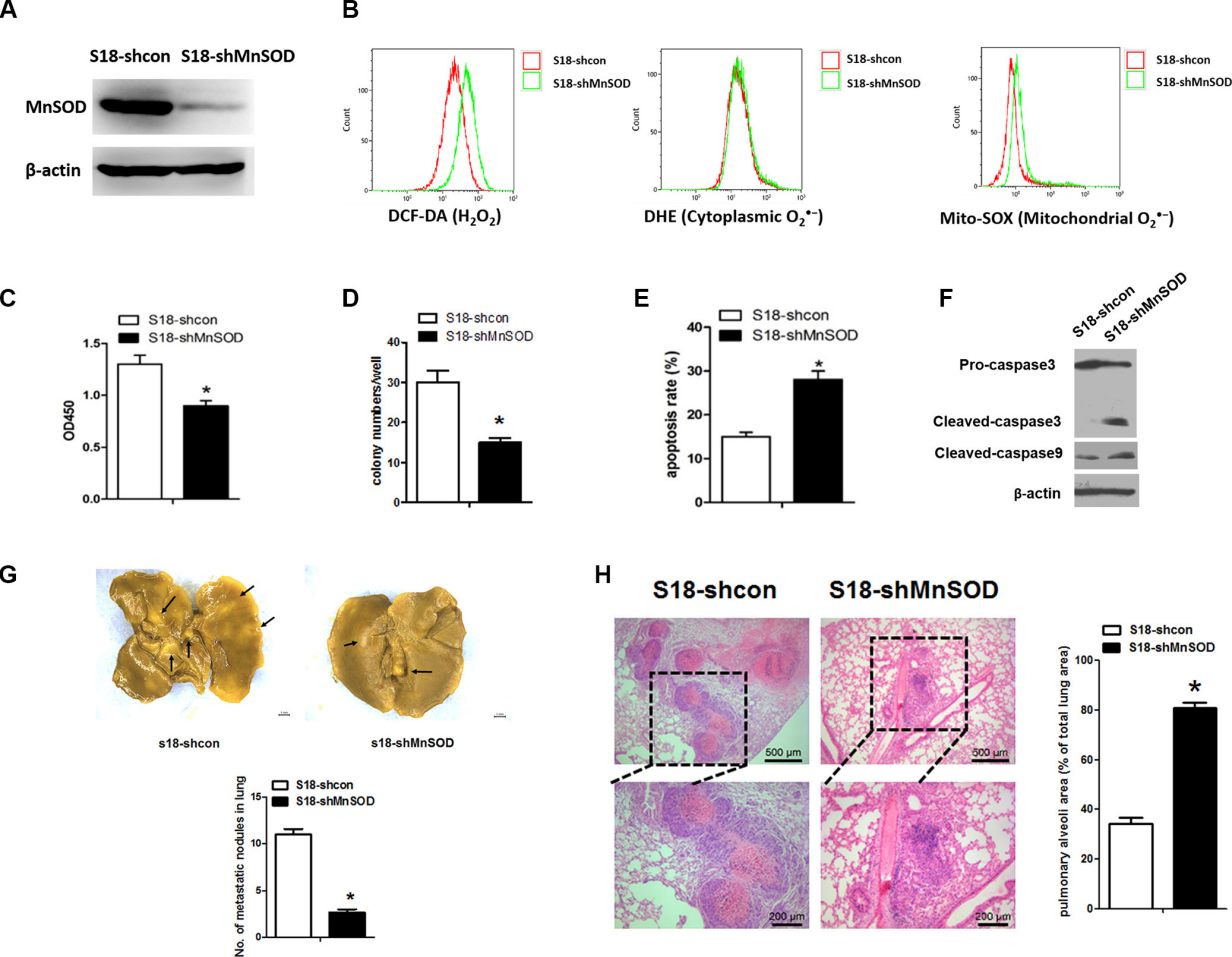
Diminished MnSOD expression promotes anoikis *in vitro* and reduces tumor formation *in vivo* (**A**) Using lentiviral transduction of shRNA, MnSOD expression was reduced in S18 cells. Western blot analysis for MnSOD and β-actin confirms the success of the established stable cell lines. (**B**) Cells were incubated for 30 min in the present of DCH-DA (10 μM), DHE (10 μM) or Mito-SOX (5 μM). Representative flow cytometry plots show the separate analysis of H_2_O_2_, cytoplasmic O_2_^·−^ and mitochondrial O_2_^·−^ content in S18-shcon and S18-shMnSOD cells under detachment cell growth condition for 24 hours. (**C**) Cell viability by CCK8 assays under suspension conditions. (**D**) Colony numbers of S18-shcon and S18-shMnSOD cells grown in soft agar. (**E**) The percent of apoptotic cells shown in response to cell culture under suspension as indicated. (**F**) Western blot analysis for markers of apoptosis (caspase 3 and caspase 9) in S18-shcon and S18-shMnSOD cells grown in suspension cultures for 24 hours was performed. (**G**) S18-shcon and S18-shMnSOD cells (2 × 10^6^ cells per injection) were injected into nude mice via tail vein (*n* = 8). Six weeks after injection, mice were killed. Representative images of shcon and shMnSOD tumors on the lungs fixed in picric acid and the average numbers of tumor nodules were shown. (**H**) Immunohistochemistry staining with hematoxylin and eosin was performed on shcon or shMnSOD lung tumor tissues, lack of tumor nodules in shMnSOD lung tissues. Scale bars represent 500 μm or 200 μm. The data are presented as mean ± S.D. For differences between two groups, the Students *t*-test was employed. *p < 0.05.

Additionally, we utilized the shRNA (shMnSOD) to develop a stably transfected S18-shMnSOD cell line (Figure 6A). Under detached conditions, there was a difference in H_2_O_2_ and mitochondrial O_2_^·−^ (but not cytoplasmic O_2_^·−^) between S18-shcon and S18-shMnSOD cells (Figure 6B). S18-shMnSOD cells also displayed increased sensitivity to anoikis and decreased cell viability following 24 hours of suspension, as compared to S18-shcon cells (Figure 6C–6E). Activation of caspase3, and 9 were involved in mitochondrial ROS-induced apoptosis (Figure 6F). To test the consequences of MnSOD loss *in vivo*, we used a nude mouse tumor model administering S18-shcon or S18-shMnSOD cells via intravenous injection to force them to grow in suspension. After six weeks, mice injected with S18-shMnSOD cells had amassed less tumor burden in the lungs compared with the S18-shcon group (Figure 6G). In addition, there were discernible differences in tumor histology when comparing lung samples from S18-shcon to S18-shMnSOD injected animals (Figure 6H). These data suggest that MnSOD is critical for the NPC colonization of lung tissue *in vivo.*

### MnSOD expression is elevated with progressing NPC tumor stage

**Figure 7:**
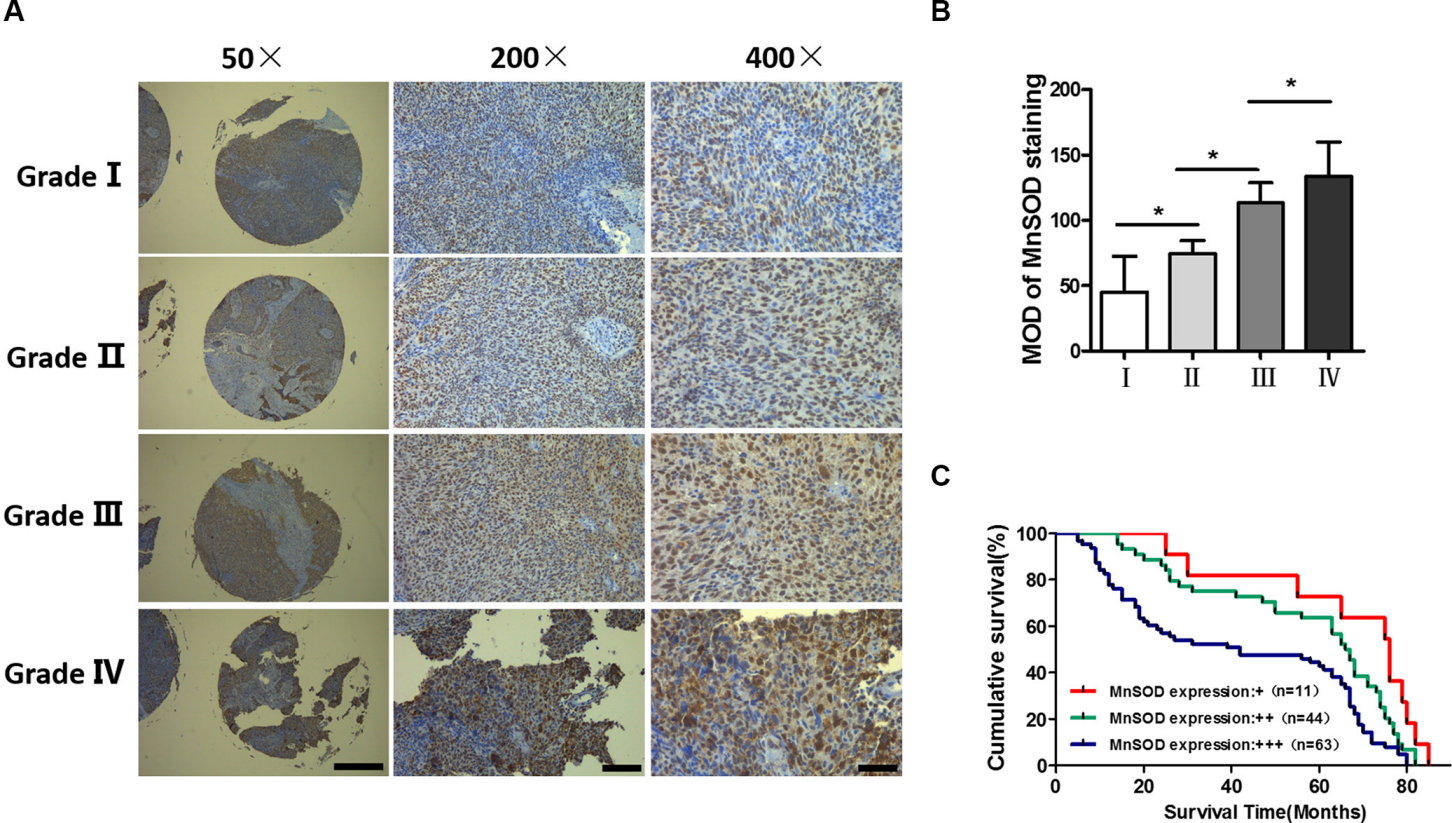
Upregulation of MnSOD in nasopharyngeal carcinoma (**A**) Representative immunostaining of MnSOD in tissue microarrays across different pathologic stages. (**B**) Quantification of MnSOD in tissue microarrays (Shanghai Outdo Biotech Co., Ltd., *n* = 118). The IHC intensity of MnSOD was analyzed using ImageJ and statistical analysis was performed using one-way analysis of variance. Error bars represent SD, *p < 0.05. (**C**) Kaplan-Meier survival curves based on MnSOD staining for patients with stage I–IV (log-rank test).

We next used immunohistochemistry to analyze MnSOD expression in tumors from NPC cancer patients (tissue microarrays, Shanghai Outdo Biotech Co., Ltd.). In NPC samples stratified by stage, MnSOD expression increased with histologic tumor grade, being highest at histologic grade IV and lowest in grade I (Figure 7A–7B). We also examined MnSOD expression in NPC samples with known clinical outcomes. In a Kaplan-Meier model, MnSOD protein expression was a strong predictor of poor survival rate, *P* = 0.0015 (Figure 7C). Correlations between MnSOD expression and various clinic pathological features are summarized in Table 1. MnSOD expression did not correlate with sex; however, it was closely associated with tumor metastasis. These results show that MnSOD expression is elevated with progressing tumor stage and correlated with poor survival, suggesting an association between high MnSOD expression and tumor aggressiveness in human NPC.

**Table 1:** Correlation between MnSOD expression and various clinical pathological features

Variables	No.of cases (*n* = 118)	Expression of MnSOD			*P*
		+ (*n* = 11)	++ (*n* = 44)	+++ (*n* = 63)	
Gender					
Male	87	8	32	47	0.974
Female	31	3	12	16	
Pathology grading					
I	5	3	1	1	0.000
II	42	6	29	7	
III	40	1	9	30	
IV	31	1	5	25	
Primary tumor					
T1	9	3	3	3	0.004
T2	43	4	24	15	
T3	30	2	9	19	
T4	36	2	8	26	
Lymph node metastasis					
N0	40	5	17	18	0.002
N1	45	2	23	20	
N2	28	2	3	23	
N3	5	2	1	2	
Distant metastasis					
M0	105	10	43	52	0.046
M1	13	1	1	11	

Correlations between MnSOD staining index scores and other categorical factors were analyzed using the Pearson chi-square test of independence.

## DISCUSSION

Anoikis is apoptosis induced by cell detachment from the extracellular matrix is a phenomenon [22]. Tumor cells need anoikis resistance to survive before metastasis, making it an essential trait of malignancy [8]. However, the mechanisms underlying anoikis resistance remain poorly understood in nasopharyngeal carcinoma (NPC) cells, which have a high rate of metastasis. In the present study, we found that the highly metastatic NPC cells, S18, have an advantage in anchorage-independent cell growth and exhibit strong anoikis resistance when compared with the poorly metastatic S26 NPC cell line (Figure 1). These results suggest that anoikis resistance is a critical feature of highly aggressive NPC cancer cells.

When adherent cells detach from the extracellular matrix (ECM) it causes metabolic defects, including marked increases in the amount of reactive oxygen species (ROS) [10]. Although low levels of ROS regulate cellular signaling and play a role in normal cell proliferation, recent studies have shown that excessive amounts or persistent elevation of ROS might lead to increased anoikis. Neutralization of ROS by antioxidant enzymes or NAC enhances survival of breast cancer cells when deprived of ECM [10, 11]. Considering the importance of ROS and their implications in anoikis, as anticipated, we found that the highly metastatic S18 cells have lower intracellular ROS production. Specifically, S18 cells have low mitochondrial O_2_^·−^and H_2_O_2_ but not cytoplasmic O_2_^·−^, when compared with S26 cells (Figure 2). These results suggest that the level of ROS is inversely correlated with malignant cell viability during the detachment process.

MnSOD is an antioxidant mitochondrial matrix protein that catalyzes O_2_^·−^ into H_2_O_2_ [23, 24]. Our data show that the highly metastatic S18 cell line display higher MnSOD protein and activity levels compared to the S26 cell line, when cells are grown in suspended conditions. These results are consistent with other reports, which found that induction of MnSOD by detachment allows cells to survive longer in suspension and depletion of MnSOD sensitizes cells to anoikis [15]. However, SOD converts superoxide radical into hydrogen peroxide may establish a steady flow of H_2_O_2_ originating from mitochondria. Interestingly, MnSOD can promote protein expression level of catalase thus would be unlikely to yield more H_2_O_2_ (Figure 4). Futhermore, though we observed Cu/Zn-SOD (a cytosolic superoxide dismutase [25]) was highly expressed in both S18 and S26 cells, it was not induced by detachment from the ECM in the first 24 hours (Figure 3). These results indicate that MnSOD, but not Cu/Zn-SOD contributes to maintaining mitochondrial bioenergetics and promoting anoikis resistance in NPC.

To further confirm the role of MnSOD in anoikis resistance, we found that the anchorage-independent growth ability of S18 cells is inhibited upon MnSOD knockdown, while growth is enhanced with MnSOD overexpression in S26 cells (Figure 4). These findings suggest that increased MnSOD activity contributes to anoikis resistance, which is a critical feature of highly aggressive NPC cancer cells. These results are strongly supported by our *in vivo* studies, which showed that the lung tumor burden in mice injected with MnSOD-deficient cells is reduced (Figure 6G).

Clinically significant elevations in MnSOD expression are associated with increased tumor invasion and metastasis in certain cancer types including gastric and esophageal cancer, breast cancer, lung carcinoma [26–29]. However, the role of MnSOD as a primary participant in the malignant transformation process in NPC cancer cells has been not investigated. Our clinical studies demonstrated that MnSOD was expressed at increased levels with progressing tumor stage, indicating an association between high MnSOD expression and tumor aggressiveness. Moreover, high expression of MnSOD correlates with extremely poor survival rates (Figure 7 and Table 1).

Another implication of the data presented here is that activation of β-catenin plays a role in the anoikis resistant of NPC. We found that the β-catenin levels were substantially decreased following matrix detachment in poorly metastatic NPC cells. Upon cell detachment, β-catenin accumulates and translocates to the nucleus, as shown with immunofluorescent staining of highly metastatic S18 cells (Figure 5B). Knockdown of β-catenin blocks MnSOD expression and induces S18 cells to become sensitive to apoptosis in suspension culture. Furthermore, overexpression of β-catenin increases MnSOD expression and represses anoikis in S26 cells. These results support an emerging role for β-catenin in regulating MnSOD in NPC, thereby making it an attractive therapeutic target to prevent tumor metastasis. Even though we document the properties of β-catenin/MnSOD in anoikis resistance, the precise mechanism by which β-catenin is activated in suspended cells and how β-catenin is involved in the regulation of the MnSOD warrants further investigation.

In summary, we established the critical role of MnSOD in conferring mitochondrial O_2_^·−^ and H_2_O_2_ degradation during anoikis resistance. Activation of β-catenin up-regulates MnSOD and might be the underlying molecular mechanism that defines the aggressive form of NPC. Thus, β-catenin could potentially be exploited as a therapeutic target to induce anoikis in circulating tumor cells and prevent metastatic growth.

## MATERIALS AND METHODS

### Cells culture and generation of stable cell lines

S18 and S26 were graciously provided by Professor Qian (Department of Nasopharyngeal Carcinoma, Sun Yat-sen University Cancer Center, Guangzhou, China). Cells were maintained in RPMI 1640 supplemented with 10% FBS. Penicillin and streptomycin were added to all cultures. Puromycin (0.5 ug/mL) was used for the generation of stable cell lines.

### Cell viability assays

Cells were plated at a density of 20,000 cells per well in 48-well or 150,000 cells per well in 6-well poly-HEMA-coated (or normal) plates. Cell viability was measured using the CCK8 assay (Dojindo, Japan) according to the manufacturer's protocol. For cell counting, detached cells were stained with trypan blue and placed in a hemocytometer.

### Soft agar colony assays

A total of 20,000 cells were suspended in RPMI 1640 (10% FBS) containing 0.7% agarose and layered on solidified medium containing 1.2% agarose in six-well plates. After solidification, the top layer was covered with the medium. When necessary, 1 mM NAC and 30 μM H_2_O_2_ was added to each layer and the cover medium. The medium was replaced every week. Images were taken after 3–4 weeks and analyzed with ImageJ software.

### Acini formation assays

12-well plates were initially coated with 30 ul 100% Matrigel and allowed to solidify, forming a gelled bed of basement membrane measuring approximately 1 mm in thickness. Cells (1 × 10^4^/ml) were seeded onto this bed as a single-cell suspension in RPMI 1640 (10% FBS) containing 2% Matrigel. The medium was replaced every 4 days. Images were obtained using a confocal laser scanning microscope after 2 weeks.

### Anoikis assays

Apoptosis was assessed by the AnnexinV/PI detection. Cells (4 × 10^5^) were plated onto poly-HEMA-coated six-well plates in growth medium to prohibit attachment. After 12, 24, and 48 h in suspension, cells were harvested, clumps were separated by trypsin (0.05%) and a mesh screen process utilized to prepare monoplast suspensions which were stained per the manufacturer's recommendation. Experiments were repeated three times.

### ROS assays

Cells were plated at a density of 200,000 cells per well in 6-well poly-HEMA-coated (or normal) plates. After 12 h and 24 h, H_2_DCF-DA (sigma), dihydroethidium (Life Technologies) or MitoSOX (Invitrogen) was added to each well at a concentration of 10 μM, 10 μM or 5 μM, respectively. After 30 min, the samples were run on the flow cytometer to detect fluorescence. Each sample was collected using 20,000 events. Amplex^®^ Red Hydrogen Peroxide/Peroxidase Assay Kit (Invitrogen, A22188) was also used to detect H_2_O_2_ according to the manual operation.

### Superoxide dismutase (SOD) activity

SOD activity was measured using a Cu-Zn/Mn-SOD assay kit (WST) (Beyotime Institute of Biotechnology, Jiangsu, China). Briefly, total SOD activity was measured by reduction rate inhibitions of 2-(4-iodophenyl)-3-(4-nitrophenyl)-5-(2,4-disulfophenyl)-2H-tetrazolium and monosodium salt (WST-1). MnSOD activity was measured by adding 10 mM potassium cyanide to inactivate Cu-Zn/SOD activity. The difference between total SOD and MnSOD activity was considered as the Cu-Zn/SOD activity. SOD activity was expressed as units/mg of protein (one unit was defined as the amount of enzyme that inhibited WST-1 reduction by 50%).

### RNA isolation and quantitative RT-PCR

Total RNA isolation from cells grown under attached and suspended conditions was carried out with TRIzol (Invitrogen, Grand Island, NY, USA), following manufacturer's protocol. 500 ng total RNA was used for reverse transcription and quantitative real-time PCR analysis (qRT-PCR). Relative mRNA quantities were determined using the comparative cycle threshold (ΔΔCt) method. β-actin was used for normalization. The primers used for *MnSOD*, *Cu, ZN-SOD*, and *ACTB* are as follows: *MnSOD* forward, 5′-CTG TTG GTG TCC AAG GCT CA-3′, *MnSOD* reverse, 5′-GTA GTA AGC GTG CTC CCA CA-3′. *Cu, Zn-SOD* forward, 5′-TGG TTT GCG TCG TAG TCT CC-3′, *Cu, Z-SOD* reverse, 5′-CTT CGT CGC CAT AAC TCG CT-3′, *ACTB* forward, 5′-GCA CTC TTC CAG CTT CCT T-3′, *ACTB* reverse, 5′-GTT GGC GTA CAG GTC TTT GC-3′. The experiments were performed in triplicate.

### siRNA transfection and short hairpin RNA

To establish stable cells expressing either MnSOD short hairpin RNA(shRNA), S18 cells were transfected with Lentiviral RNAi system (The lentivirus skeleton plasmid were gifts from Prof. Peng Xiang (Sun Yat-sen University, China) and selected by 0.5 ug/mL puromycin (Invitrogen, Carlsbad, CA, USA) for 15 days. For siRNA knockdown, MnSOD siRNA and a control siRNA were purchased from RiboBio. According to the manufacturer's instructions, transfections were performed at approximately 60% confluency using HiPerFect Transfection Reagent (Qiagen).

### Western blotting

Cells were harvested and lysed for total protein extraction. Protein concentration was determined using Bio-Rad DC protein assay kit (Bio-Rad Laboratories) according to manufacturer's protocol. Aliquots of equal amounts of protein from the cell lysates were subjected to Western blot analysis. Antibodies used include those specific for MnSOD(BD Biosciences), catalase (proteintech), caspase3 (Cell Signaling Technology), caspase9 (Cell Signaling Technology), β-catenin (Cell Signaling Technology) and β-actin (Sigma, MO, USA).

### Animal experiments

Male athymic mice between 5 and 6 weeks of age were obtained from Shanghai Institutes for Biological Sciences (Shanghai, China). 2 × 10^6^ cells were injected into the lateral tail vein of 7-week-old nude mice. Six weeks after injection, the mice were sacrificed, and lungs were removed. Lungs were fixed in Bouin's solution for 24 hours and stored in 70% ethanol before analysis. All the animal procedures were outlined in the guidelines of Institutional Animal Care and Use Committee at SYSU.

### Immunofluorescence

S18 and S26 cells plated on culture slides or smear after the suspension culture were fixed in ice-cold 4% paraformaldehyde. Then, samples were permeabilized for 20 min with 0.01% Triton X-100 in PBS and blocked with normal goat serum at 37°C for 1 hour. After washing 3 times with PBS, they were incubated with β-catenin (Cell Signaling Technology) overnight at 4°C, then were followed by fluorescent secondary antibodies (1:200 dilution in blocking buffer, 1 h, room temperature). After immunolabeling, cells were washed, stained with 4, 6-di-amino-2-phenylindole (DAPI) (Sigma, St. Louis, MO, USA) for 5 min at room temperature. Cells were visualized under a confocal microscope (Axio Observer Z1, ZEISS, Jena, Germany).

### Immunohistochemistry

Tissue microarray (Shanghai Outdo Biotech Co., Ltd., *n* = 118) were deparaffinized and dehydrated with graded alcohol. The samples were pretreated with 0.01 M citrate buffer (pH 6.0) for 2 min at 100°C in an autoclave; then the slides were allowed to cool to room temperature. Endogenous peroxidase activity was quenched by incubation in methanol containing 3% H_2_O_2_ for 10 min at room temperature. After several washes in Phosphate-Buffered Saline Tween-20 (PBST) (pH 7.2), the sections were blocked with goat serum for 60 min at room temperature and then incubated with MnSOD antibody (BD Biosciences) overnight at 4°C in a humidified chamber. After a brief rinse in PBST, sections were incubated for 40 min at 37°C with a biotin-conjugated secondary antibody (mouse) followed by incubation with DAB for 15 s. After rinsing with distilled water, sections were counterstained with hematoxylin. As a negative control, slides were incubated in PBS in place of primary antibody. The IHC intensity of MnSOD was analyzed using ImageJ.

### Statistical analyses

Data are presented as means ± SD. The difference between experimental groups was assessed by ANOVA or two-tailed paired Student's *t*-test. The log-rank test was used for survival analysis. A value of *p* < 0.05 was considered statistically significant.
